# Human Carrying Capacity and Human Health

**DOI:** 10.1371/journal.pmed.0010055

**Published:** 2004-12-28

**Authors:** Colin D Butler

## Abstract

The issue of overpopulation has fallen out of favor among most contemporary demographers, economists, and epidemiologists. Discussing population control has become taboo. This taboo could be hazardous to public health

The issue of human overpopulation has fallen out of favor among most contemporary demographers, economists, and epidemiologists. Discussing population control has become a taboo topic. Yet, this taboo has major implications for public health.

The silence around overpopulation prevents the global health community from making the necessary link between the planet's limited ability to support its people (its carrying capacity—see sidebar on following page) and health and development crises. In this article, I describe how popular thinking on population control has been shaped over the last 200 years, and how our failure to address the population explosion may be one cause of recent epidemics and social unrest.

## Overpopulation Concerns Peak, Then Decline

The question of human overpopulation and its relationship to human carrying capacity has been controversial for over two centuries. In 1798 the Reverend Thomas Malthus put forward the hypothesis that population growth would exceed the growth of resources, leading to the periodic reduction of human numbers by either “positive checks”, such as disease, famine, and war, or “preventive checks”, by which (in the absence of contraception) Malthus meant restrictions on marriage. This “Malthusian view” was rapidly accepted by most politicians, demographers, and the general public, and remained popular until fairly recently.

Malthus's worst fears were not borne out through the century following his death in 1834—food production largely kept pace with the slowly growing global population. However, soon after 1934, the global population began to rise steeply as antibiotics, vaccines, and technology increased life expectancy. By the 1960s, concerns of a mismatch between global population and global food supply peaked—expressed in books such as Paul Ehrlich's 1968 *The Population Bomb* [1]. This book predicted a future scarred by increasing famine, epidemic, and war—the three main Malthusian positive checks.

In 1966, United States President Lyndon Johnson shipped wheat to India to avert a famine on the condition that the country accelerate its already vigorous family planning campaign [2]. Johnson was part of an unbroken series of US presidents concerned with the harmful effects of rapid population growth in developing countries. This line extended (at least) from John F. Kennedy to Jimmy Carter. George H. W. Bush was also sympathetic to this view, prior to becoming vice president in 1981.

But the 1970s surprised population watchers. Instead of being a period shadowed by calamitous famine, the new crop strains introduced by the “Green Revolution” (especially grains such as rice, wheat, and maize) caused a dramatic increase in the global production of cereals, the main source of energy in the global diet. Among the development community, despair turned into cautious optimism. By the end of the decade, the public health community felt sufficiently empowered to proclaim “Health for All by the Year 2000”. Average life expectancy continued to zoom upwards almost everywhere—even in sub-Saharan Africa.

The introduction of safe contraception contributed to a rapid fertility decline in many countries. But while the rate of global population growth declined from its peak in the late 1960s, the absolute increment of increase in annual global population continued to grow. Most population-related scientists, including food scientists and demographers, as well as US President Jimmy Carter, continued to be very concerned about global overpopulation. In 1970, the father of the Green Revolution, the agricultural scientist Norman Borlaug, was awarded the Nobel Peace Prize. In his Nobel lecture, Borlaug warned that the success of the Green Revolution would buy a breathing space for humankind of three decades, unless equivalent action was taken to reduce fertility rates [3]. China tightened its fertility policy in this decade, introducing its one-child policy in 1979.[Fig pmed-0010055-g001]

**Figure pmed-0010055-g001:**
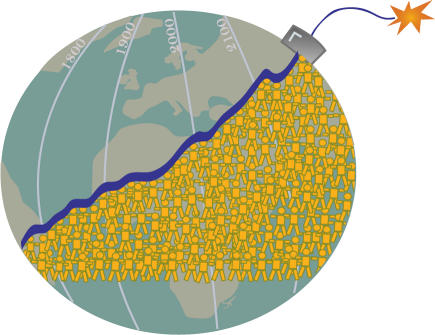
We are failing to confront the population explosion (Illustration: Sapna Khandwala)

## Concern for the Third World Fades

With hindsight, the 1970s can be seen as the decade when widespread concern about overpopulation started to fade. The social and economic milieu of many developed countries, especially in the US, started to change. US foreign aid, as a percentage of the gross national product, declined from the late 1960s, perhaps in part because of the competing needs of the Vietnam War but also perhaps because of the apparent success of development in the Third World. The economic policies known as Keynesianism, which had been dominant since the end of World War II in many developed nations, came under sustained attack. These policies had placed a high value on full employment and social security. Keynesian policies restrained domestic inequality through high taxation and the promotion of social norms that censured conspicuous consumption (such as company executives exercising restraint in their personal salaries and people buying small houses). Shortly before his death, J. M. Keynes had also been crucially involved in the establishment of the World Bank. Keynes appears to have been personally committed to the advance of global justice, and to the reduction of inequality both within and between nations [4].

The world oil shock in 1973 contributed both to “stagflation”—a combination of rising unemployment with higher prices—and to increased economic power for the oil-producing countries of the Third World. Indeed, the term “Third World” came to be considered pejorative and was replaced by the “South”. Stagflation was interpreted as a failure of Keynesian policy. The demise of Keynesianism was accompanied by a further decline in concern for Third World development among elite economists and the general public.

It is unlikely that the issue of global population policy figured into the election that put US President Ronald Reagan into office in 1980. Nevertheless, Reagan's policies were to cement a new orthodoxy about global overpopulation and development strategies. Unlike his republican predecessor, Richard Nixon, Reagan considered concerns about global population size to be “vastly exaggerated” [5]. In the same year, the US surprised the family planning world by abdicating its previous leadership in the effort to promote global family planning, at the International Conference on Population, held in Mexico City in 1984. The US took this position against the strenuous opposition of the Population Association of America, which represented many US demographers [5].

As foreign aid budgets fell, the “Health for All” targets began to slip from reach. Instead, international agencies promoted structural adjustment programs, health charges for patients (“user fees”), and the “trickle down” effect as the best ways to promote development. It is plausible that a fraction of the public who remained concerned about Third World development thought that these new economic policies deserved a chance. Less charitably, the new economic policies also appeared to allow people already financially comfortable to abdicate concern for Third World development because the new orthodoxy asserted that market deregulation, rather than aid, was the royal road to development. The increased domestic inequality of recent decades in developed countries [6] probably also contributed to a reduction in concern for the Third World, as working people have had to struggle harder to keep their position in their own society.

It is now clear that market deregulation and generally high birth rates have proven disastrous in many Third World countries. “Health for All”, if recalled at all, is now seen as absurdly optimistic. The failure of development is most obvious in many sub-Saharan countries, where life expectancy has fallen substantially. But life expectancy has also fallen in Haiti, Russia, North Korea, and a handful of other nations [7]. The causes for this decline in life expectancy are multiple and complex. Causes that are usually listed include HIV/AIDS (Zimbabwe and Haiti) [8,9], ethnic hatred (Rwanda) [10], crop failure (North Korea) [11], poor governance and poverty (several parts of Africa) [12], and alcoholism (Russia) [13].

Causal theory is complex. Every cause has a cause, and, increasingly, causes are being considered as a part of causal chains, causal webs, and causal snowballs. Some theorists distinguish between identifiable “proximal” causes and deeper, underlying, or “distal”, causes [14]. Yet, among the multitude of causes that can be identified for declines in either total population or life expectancy, overpopulation is hardly considered, except by dissident public health workers such as Maurice King [15]. Demography, the discipline that would appear to be the most likely holder of the Malthusian baton, is now almost entirely silent about overpopulation in developing countries [16]. Instead, most mainstream demographers appear to consider population ageing and European underpopulation as the most important demographic issues for this century. On the other hand, the role of the rapid demographic transition in China (from large to small families, with an average of two or fewer children) is rarely credited as central to the Chinese economic miracle.

## Overpopulation: A Cause of Crises in Africa?

Often, the carrying capacity of one region at one point in time is boosted by the appropriation of the carrying capacity from other people and even other generations. Such resources include oil, deep sea fish, and the stability of the global climate and ecological systems. But in Rwanda, the most densely populated country in Africa, the importation of such resources has long been limited. Unlike other densely populated countries such as Hong Kong and Holland, Rwanda's economy at the time of its most infamous genocide, in 1994, depended almost exclusively on its primary production [17]. The country had little industry, few exports, and little tourism. The price of its most important export, coffee, had declined steeply just before the genocide [18]. Unlike many Asian countries, Rwanda also received few remittances from Rwandans working as guest workers abroad [17].

Among the many different explanations for the horrific 1994 Rwandan genocide, the possibility of a Malthusian check (also called “demographic entrapment”) is scarcely mentioned [17,19]. A Malthusian check in Rwanda was plausible not only because the total population was too large, but perhaps more importantly because the rate of population growth in Rwanda was faster than the capacity of Rwandan society to process the additional people. As a result, many indicators of development went backwards. The limited agricultural capacity forced many young men into Kigali, causing a concentration of young men with few prospects other than what they might gain from violence.

There is even less scientific discussion that entertains the possibility that the sub-Saharan epidemic of HIV/ AIDS may also be a Malthusian check [19]. This is plausible if one applies a conceptual framework that combines the erosion of human carrying capacity through the same rapid population growth seen in Rwanda, with a consequent decline in per capita income and food supply. Furthermore, slowly operating feedbacks occurring as a result of the epidemic further undermined development, including the loss of human capital as teachers died [20], the loss of agricultural expertise as farmers died [21], and a deepening debt and loss of productivity from the countless funerals. And leaders in the developed world and many within Africa itself failed to devote the resources and provide the leadership required to quell the epidemic.

## Conclusion

Maurice King refers to the silence on overpopulation as the “Hardinian Taboo”, named after the American ecologist Garett Hardin, who described the taboos that humans use to avoid confronting the need for population control [22]. Daniel Orenstein, at the Center for Environmental Studies at Brown University, has argued that powerful social norms inhibit debate about overpopulation in one of the world's most intractable trouble spots, Israel and Palestine [23].

Whatever the cause of the scarcity of modern academic analysis, the related issues of human carrying capacity and overpopulation deserve fresh consideration. The entrapment model has an explanatory power that is lacking in more superficial causal explanations. Of course, solving entrapment is very difficult, but as with most medical problems, a proper diagnosis will help identify the proper treatment.

**Human Carrying Capacity**

Human carrying capacity is the maximum population that can be supported at a given living standard by the interaction of any given human-ecological system. This apparently simple concept has many nuances and is rarely used by population scientists. However, in rejecting this term, purists risk making a terrible conceptual flaw, that of thinking that environmental and human resources are largely irrelevant to human population size.

It is irrefutable that human ingenuity and cooperation can increase human carrying capacity [24]. But even so, human welfare will continue to depend on the external world, including for resources such as food and water. Humans are neither computer ciphers nor caged mice. That is to say, while a given area might tolerate a theoretically higher density of human population than it does, the reality of human evolution in distinct groups, separated by culture, religion, and language, means that this theoretical maximum will rarely be attained. A degree of underused carrying capacity can be viewed as a desirable buffer around disparate groups, vital for reducing tension and preventing conflict.

Even culturally homogenous groups can outgrow their carrying capacity, as in the case of the Great Hunger in Ireland in the 1840s, when the population crashed because of famine, disease, and emigration. Indeed, Malthusian theory was used, in part, to justify the scanty aid provided to the Irish from Britain, a country that did not identify closely with the Irish.
